# Genotypic diversity of the Helicobacter pylori vacA c region and its correlation with gastric disease outcomes

**DOI:** 10.1099/jmm.0.001969

**Published:** 2025-03-07

**Authors:** Mounia El khadir, Souad Oirdi Zahir, Samia Alaoui Boukhris, Dafr-Allah Benajah, Sidi Adil Ibrahimi, Laila Chbani, Mohamed El Abkari, Bahia Bennani

**Affiliations:** 1 Laboratory of Human Pathology Biomedicine and Environment, Faculty of Medicine, Pharmacy and Dentistry of Fez, Sidi Mohammed Ben Abdellah University, Fez, Morocco; 2 The Higher Institute of Nursing Professions and Health Techniques, Fez, Morocco; 3 Department of Hepato-Gastroenterology, Hassan II University Hospital Center, Fez, Morocco; 4 Department of Pathological Anatomy, Hassan II University Hospital Center, Fez, Morocco

**Keywords:** gastric diseases, genetic diversity, *Helicobacter pylori*, * vacA* c

## Abstract

**Introduction*****.** Helicobacter pylori* infection is a major cause of peptic ulcer and gastric adenocarcinoma. The infection progression to severe diseases depends on several factors, including bacterial ones. CagA and VacA are two major virulence factors widely studied and implicated in *H. pylori* diseases.

**Gap statement.** Although the allelic prevalence of the s, m, i and d regions of the *H. pylori vacA* gene and their relation with gastric disease outcomes have been largely studied, there is a gap in knowledge regarding the prevalence and the association of the *vacA* c region with those pathologies. This polymorphic region could exhibit a large variability which might impact the virulence of the bacterium and the severity of gastric damage.

**Aim.** The aim of this study was to investigate the prevalence of *H. pylori vacA* c alleles and to assess the association of *vacA* mosaicism with gastric damage in a large Moroccan population.

**Methodology.***H. pylori* positive gastric biopsies, obtained in 709 consenting patients who consulted the gastroenterology department of the Hassan II University Hospital of Fez (Morocco) for clinical endoscopy, between 2009 and 2019, were used in this study. DNA extracted from the biopsy samples was used to determine the *vacA* c genotype using specific PCRs. *H. pylori cagA*, *vacA* s, *vacA* m, *vacA* d and *vacA* i genotypes of these samples were previously determined.

**Results.** The *vacA* c2 genotype was detected in 44.7% of samples and the *vacA* c1 genotype in 16.5% of cases. Multiple infections (detection of the two *vacA* c allelic forms) were obtained in 9% of samples. Correlation of *H. pylori vacA* c genotype with pathologies showed that the *vacA* c1 allele was strongly associated with the risk of gastric cancer (GC) [odds ratio=3.14, confidence intervals 95% (1.08–9.09)].

**Conclusion.** The results of this study confirm that despite the large *H. pylori* genetic diversity, the genotypic distribution is marked by a predominance of the less virulent strains in Morocco. Also, even if *vacA* allelic combinations seem to impact the toxicity of this bacterium, patients infected with *H. pylori vacA* c1 genotype are more likely to develop GC than those infected by *H. pylori vacA* c2 genotype.

## Introduction

*Helicobacter pylori* is a strictly human pathogen that infects more than half of the world’s population. Due to its ability to resist gastric acidity, this bacterium settles in the human stomach, causing severe gastroduodenal diseases, notably chronic gastritis, peptic ulcer, MALT (Mucosa-Associated Lymphoid Tissue) lymphoma and gastric adenocarcinoma [1]. The gastric cancer (GC) is the third leading cause of cancer deaths worldwide [2]. The *H. pylori* infection evolution is influenced by the environment, the host and the high genetic diversity of the bacteria. Among the genetic factors contributing to the pathogenesis, *cagA* and *vacA* are two genes of *H. pylori* that have been widely studied.

The *cagA* gene is harboured in the cag pathogenicity island (*cag*PAI) and encodes a 120–145 kDa immunodominant protein that is injected into the host cells via the type IV secretion system. Thus, it plays an important role in the infection process.

The *vacA* gene encodes an 88 kDa effector protein composed of two subunits. The protein’s diversity includes variations in its regions, influencing its pathogenicity and interaction with host cells. The *vacA* gene has five variable regions; the signal region ‘s’ may exist as active allele s1 or s2, encoding the signal peptide located at the N-terminal of the VacA, and is crucial for the toxin’s secretion [3]. In the mid-region ‘m’ with its two allelic forms, m1 or m2, the encoding gene is capable of binding to a wide range of cells, thus causing different levels of toxicity (high level for m1 or much less for m2) [4]. The intermediate region ‘i’ is represented by three subtypes (i1, i2 and i3) [5] and considered to be a better predictor of the disease’s severity than the ‘s’ or the ‘m’ region. In fact, the subtype *vacA* i1 was significantly associated with different gastric pathologies according to different geographical areas. It was associated with GC in an Iranian study [6], gastric ulcers in Iran [7] and duodenal ulcers (DUs) in Italy [8]. The *vacA* d region, previously classified into 2 genotypes (d1: without deletion and d2 with 81 bp deletion) [9], was recently reclassified into 31 genotypes. In fact, according to a recent bioinformatics study, this region presented a high degree of polymorphism and diverse genetic variations (located between amino acids 332 and 367 in the *H. pylori* strain 60, 190). These 31 genotypes have been classified into three main types: K-type, Q-type and E-type (each one contains several subtypes). The K-type seems to be the most virulent and is notably related to GC [10]. The *vacA* d1 genotype has been reported to be significantly more prevalent in gastric adenocarcinoma and/or peptic ulcer disease than in gastritis [11]. Finally, the *vacA* c region, also existing as c1 (with 15 bp deletion) or c2 (without deletion) genotypes, is recently determined as a polymorphic site in the 3′ end of *vacA*, but its biological role in vacuolating activity and pathogenicity is still not well known [11]. Some studies reported that the *vacA* c genotype represents a strong predictor of GC.

The aim of this study was to determine, for the first time, the prevalence and the distribution of the *H. pylori vacA* c alleles and to assess the association between *vacA* mosaicism and gastric damage in a large Moroccan population.

## Methods

### Patients and sampling

Biopsies obtained in 709 consenting patients who consulted the gastroenterology department of the Hassan II University Hospital of Fez (Morocco) for clinical endoscopy, between 2009 and 2019, and who were *H. pylori* positive were used in this study. Two gastric biopsy samples of each patient, stored at −80 °C in PBS solution, were ground and used for DNA extraction. In total, 120 µl of cell lysate was treated with 40 µl of proteinase K (200 µg ml^−1^ in 3% Triton X-100) overnight at 37 °C [12]. Proteinase K was inactivated by heating at 92 °C for 10 min, and the supernatant was purified by the phenol/chloroform method [13]. The obtained DNA concentration is between 167 and 250 ng µl^−1^. *β*-globin PCR was performed using primer sets (GH20/PC04) to check the DNA quality [14], and PCR of the *glmM* gene was performed on all extracted samples to confirm the presence of *H. pylori* DNA. Biopsies from these patients previously used for the identification of *cagA*, *vacA* s, m and i genotypes [15,16] were used to determine the allelic distribution of the *vacA* c and to evaluate its association with gastroduodenal pathologies. This study was approved by the Institutional Review Board of the Hassan II University Hospital of Fez, Morocco.

Participants were aged between 18 and 99 years (±15 369) and they were 351 (49.5%) men and 358 (50.5%) women. Among them, 51.6% were aged<50 years old. Fibroscopic and histopathological exams showed that 66.9% of them had gastritis, 15.7% had a DU, 9.3% had a gastric ulcer, 6.9% had a GC and 1.3% had gastroduodenal ulcer. In addition, 70.1% (497/709) of *H. pylori* biopsy samples carried the *cagA* gene, as previously described [16].

### *vacA* c genotyping

The *vacA* c region was determined by single PCR on extracted DNA using degenerated primers to amplify c1 (deletion of 15 bp) and c2 (no deletion) regions separately, as described previously [11]. These primers delimited a region of 600–700 bp. Since the size of both amplicons that will be generated (for *vacA* c1 and *vacA* c2 regions) ranges from 600 to 700 bp with a difference of only a 15 bp deletion, PCRs were performed separately for each genotype.

The reactions were performed in a total volume of 25 µl each, containing 1×Dream *taq* buffer, 25 mM MgCl_2_, 200 µM of each dNTP (deoxyribonucleotide triphosphate), 10 µM of each primer, 2U of dream *taq* DNA polymerase and 3 µl of purified DNA. Positive and negative controls were used for each reaction. The PCR conditions were as follows: 96 °C for 3 min and then 35 cycles of 96 °C for 40 s, optimized annealing temperature at 55.5 °C for 40 s, extension at 72 °C for 40 s and a final extension at 72 °C for 7 min. Finally, amplified PCR products were resolved in 1.5% agarose gel, stained with ethidium bromide and visualized under a UV light source.

### Data analysis

Statistical analysis was performed using Statistical Product and Services Solutions (version 20, SPSS Inc., Chicago, Illinois, USA) software. The frequencies of each allele and genotype combinations were calculated. Fisher’s exact or Chi-square tests were used to evaluate the association between *H. pylori vacA* s, m, i and/or c region genotypes and gastric pathologies. *P*-values<0.05 were considered statistically significant. Since CG and DU were considered opposite outcomes of *H. pylori* infection, we used the DU group as reference pathology. The multivariate analysis was performed to highlight the most incriminated factors in the occurrence of GC using simple logistic regression analysis. All variables with *P*≤0.20 were included in the initial model. The final model was obtained using a stepwise elimination method to identify potential independent factor(s) associated with GC. The results were expressed as odds ratio (OR), 95% confidence intervals (CI) and *P*-values.

## Results

**Fig. 1.**
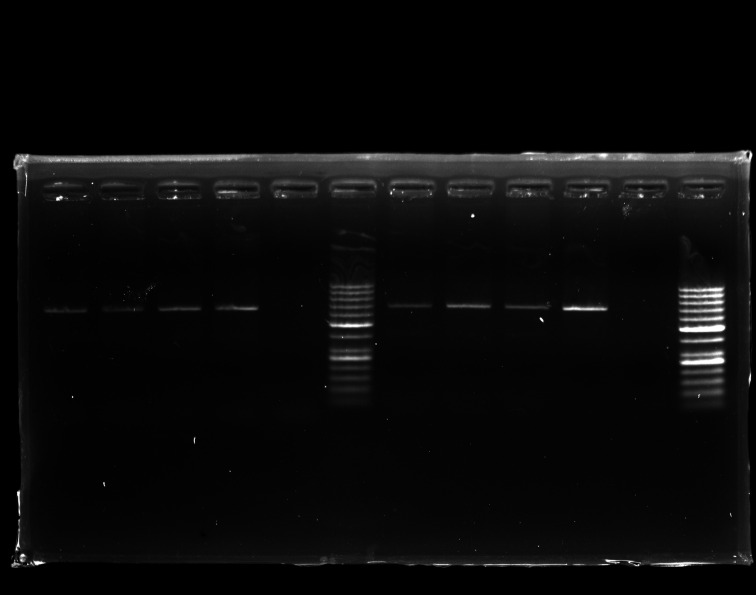
Agarose gel electrophoresis representing the *vacA* c region: 1–3: *vacA* c1 positive samples, 4: *vacA* c1 positive control, 5: negative control, 6: 50 bp ladder, 7–9: *vacA* c2 positive samples, 10: *vacA* c2 positive control, 11: *vacA* c2 negative control, 12: 50 bp ladder.

*vacA* c has been genotyped in 70.2% of cases. Molecular analysis revealed a predominance of *vacA* c2 genotype with a rate of 44.7% (317/709), compared to *vacA* c1 detected in 16.5% (117/709) of cases. Combined genotypes *vacA* c1c2 were obtained in 9% (64/709) of samples, and 29.8% (211/709) of samples were not genotyped. Fig. 1 shows the profiles of obtained amplicons using the *vacA* c primers.

The distribution of the *vacA* c region (c1 and c2) according to the other *vacA* region (s, m, i) genotypes, *cagA* status and patients’ gender and age has been studied, and the results are presented in Table 1. They show that *vacA* c1 *H. pylori* was significantly more dominant in men (59.2%) than in women (*P*=0.006). Also, *cagA* is significantly predominant in strains harbouring *vacA* c1 (*P*<0.001). The *H. pylori vacA* mosaicism study shows that most *vacA* c1 strains carried *vacA* s1, *vacA* m1 and *vacA* i1 (80.2, 89.1 and 92.7%, respectively) and that 81.2% *vacA* c1 carried *vacA* s1m1i1. Those associations were statistically significant (*P*<0.001).

**Table 1.** *vacA* c distribution among gender, age, *cagA* gene and the *vacA* s, m, i genotypes

		*vacA* c*N* (%)					
		Not genotyped	c1	c2	c1c2	Total	*P*-value
Gender	Men		61 (59.2)	125 (43.6)		186 (47.7)	0.006
	Women		42 (40.8)	162 (56.4)		204 (52.3)	
	Total		103 (100)	287 (100)		390 (100)	
Age	<50		54 (52.4)	156 (54.4)		210 (53.8)	0.41
	≥50		49 (47.6)	131 (45.6)		180 (46.2)	
	Total		103 (100)	287 (100)		390 (100)	
*cagA*	Negative		10 (9.7)	106 (37.2)		116 (29.9)	<0.001
	Positive		93 (90.3)	179 (62.8)		272 (70.1)	
	Total		103 (100)	285 (100)		388 (100)	
*vacA* s	s1		81 (80.2)	67 (23.4)		148 (38.2)	<0.001
	s2		20 (19.8)	219 (76.6)		239 (61.8)	
	Total		101 (100)	286 (100)		387 (100)	
*vacA* m	m1		90 (89.1)	36 (12.7)		126 (32.7)	<0.001
	m2		11 (10.9)	248 (87.3)		259 (67.3)	
	Total		101 (100)	284 (100)		385 (100)	
*vacA* i	i1		89 (92.7)	39 (13.8)		128 (33.9)	<0.001
	i2		7 (7.3)	243 (86.2)		250 (66.1)	
	Total		96 (100)	282 (100)		378 (100)	
*vacA* smi	s1m1i1	39 (18.7)	78 (67.8)	16 (5)	14 (21.9)	147 (20.9)	<0.001*
	s1m1i2	4 (1.9)	1 (0.9)	11 (3.5)	4 (6.2)	20 (2.8)	
	s1m2i1	3 (1.4)	0	11 (3.5)	2 (3.1)	16 (2.3)	
	s1m2i2	8 (3.8)	0	29 (9.1)	2 (3.1)	39 (5.5)	
	s2m1i1	8 (3.8)	6 (5.2)	1 (0.3)	1 (1.6)	16 (2.3)	
	s2m1i2	1 (0.5)	1 (0.9)	8 (2.5)	0	10 (1.4)	
	s2m2i1	15 (7.2)	5 (4.3)	11 (3.5)	1 (1.6)	32 (4.5)	
	s2m2i2	70 (33.5)	5 (4.3)	193 (60.9)	9 (14.1)	277 (39.3)	
	Multiple infections	29 (13.9)	14 (12.2)	32 (10.1)	29 (45.3)	104 (14.8)	
	Incomplete genotype	32 (15.3)	5 (4.3)	5 (1.6)	2 (3.1)	44 (6.2)	
	Total	209 (29.6)	115 (16.3)	317 (45)	64 (9.1)	705 (100)	

*Kruskall Wallis test, the correlation between *vacA* c and *vacA* mosaic genotypes was done only for samples with a simple infection and with complete *vacA* genotypes.

The *vacA* mosaicism shows a predominance of the *vacA* s2m2i2c2 genotype (27.2%). The incomplete genotype indicating the unsuccessful amplification of at least one of the *vacA* regions (s, m, i or c) was obtained in 27.1% of cases.

To determine the genotype–pathology association and in order to avoid statistical bias, we have excluded from the analysis (i) samples with multiple infections (MI) (multiple genotypes for the same alleles), (ii) cases with incomplete *vacA* genotype (Inc *vacA*) and (iii) gastroduodenal ulcer cases. The analysis results (Table 2) show that *H. pylori* harbouring *vacA* c1 is significantly predominant in GC cases (50%) than in the other pathologies (*P*=0.011). However, there is an equal distribution of *vacA* c1 and *vacA* c2 *H. pylori* in GC. Likewise, when studying the *vacA* combined-genotype association with pathologies, *H. pylori vacA* s1m1i1c1 and *vacA* s2m2i2c2 have an equal distribution rate in GC (30.4%) and in gastric ulcer (32.4 and 35.3%, respectively). However, *H. pylori vacA* s2m2i2c2 was significantly predominant in DU and gastritis cases (46.8 and 56.5%, respectively) (*P*=0.009). *vacA* s2m1i1c2 and *vacA* s2m1i2c1, which belong to the rare *vacA* genotypes, are exclusively present in a single case of GC, while *vacA* s1m1i2c1 is present in a single case of gastritis.

**Table 2.** Distribution of demographic and *vacA* mosaicism based on the gastroduodenal diseases

		GC	Gastric ulcer	DU	Gastritis	Total	*P*-value
Gender	Men	29 (59.2)	44 (66.7)	81 (73)	189 (39.9)	343 (49)	<0.001
	Women	20 (40.8)	22 (33.3)	30 (27)	285 (60.1)	357 (51)	
	Total	49 (100)	66 (100)	111 (100)	474 (100)	700 (100)	
Age	<50	16 (32.7)	30 (45.5)	59 (53.2)	259 (54.6)	364 (52)	0.020
	≥50	33 (67.3)	36 (54.5)	52 (46.8)	215 (45.4)	336 (48)	
	Total	49 (100)	66 (100)	111 (100)	474 (100)	700 (100)	
*vacA* c	c1	13 (50)	12 (34.3)	13 (27.1)	62 (22.3)	100 (25.8)	0.011
	c2	13 (50)	23 (65.7)	35 (72.9)	216 (77.7)	287 (74.2)	
	Total	26 (100)	35 (100)	48 (100)	278 (100)	387 (100)	
*vacA*	s1m1i1c1	7 (30.4)	11 (32.4)	13 (27.7)	45 (16.7)	76 (20.4)	0.009*
	s1m1i1c2	1 (4.3)	2 (5.9)	4 (8.5)	9 (3.3)	16 (4.3)	
	s1m1i2c1	0	0	0	1 (0.4)	1 (0.3)	
	s1m1i2c2	0	2 (5.9)	1 (2.1)	8 (3)	11 (2.9)	
	s1m2i1c2	1 (4.3)	0	1 (2.1)	9 (3.3)	11 (2.9)	
	s1m2i2c2	1 (4.3)	6 (17.6)	4 (8.5)	18 (6.7)	29 (7.8)	
	s2m1i1c1	0	0	0	5 (1.9)	5 (1.3)	
	s2m1i1c2	1 (4.3)	0	0	0	1 (0.3)	
	s2m1i2c1	1 (4.3)	0	0	0	1 (0.3)	
	s2m1i2c2	1 (4.3)	1 (2.9)	1 (2.1)	5 (1.9)	8 (2.1)	
	s2m2i1c1	1 (4.3)	0	0	4 (1.5)	5 (1.3)	
	s2m2i1c2	1 (4.3)	0	1 (2.1)	9 (3.3)	11 (2.9)	
	s2m2i2c1	1 (4.3)	0	0	4 (1.5)	5 (1.3)	
	s2m2i2c2	7 (30.4)	12 (35.3)	22 (46.8)	152 (56.5)	193 (51.7)	

*Kruskall Wallis test.

To evaluate the effects of *H. pylori* genotypes and demographic characteristics on the occurrence of GC, we have compared the results obtained in GC and DU cases. The univariate analysis (Table 3) shows that 67.3% of GC patients were aged over 50, while 53.2% of DU patients were under 50 (*P*=0.01). Moreover, a predominance of *vacA* c2 was observed in DU patients (72.9%), while a similar distribution (50%) of *vacA* c1 and *vacA* c2 was observed in GC cases (*P*=0.04). All variables with *P*<0.2 were included in logistic regression analysis, and duodenal ulcer pathology was considered a control group. The logistic regression results showed that *vacA* c1 alleles were strongly associated with the risk of GC [OR=3.14, CI 95% (1.08–9.09), *P*=0.03], while men seem to be more at risk of DU than GC [OR=0.26, CI 95% (0.08–0.79), *P*=0.01] (Table 4).

**Table 3.** Distribution of demographic and risk factors compared to DU and GC cases

		DU	GC	*P*			DU	GC	*P*			DU	GC	*P*
**Gender**	Men	81 (73)	29 (59.2)	0.06	***vacA* sm**	s1m1	30 (39.5)	15 (37.5)	0.02	***VacA* smic**	s1m1i1c1	13 (27.7)	7 (30.4)	0.41*
	Women	30 (27)	20 (40.8)			s1m2	7 (9.2)	2 (5)			s1m1i1c2	4 (8.5)	1 (4.3)	
**Age**	<50 years	59 (53.2)	16 (32.7)	0.01		s2m1	1 (1.3)	6 (15)			s1m1i2c1	0	0	
	≥50 years	52 (46.8)	33 (67.3)			s2m2	38 (50)	17 (42.5)			s1m1i2c2	1 (2.1)	0	
***vacA* s**	s1	37 (48.1)	17 (40.5)	0.27	***vacA* sc**	s1c1	15 (26.8)	7 (28)	0.004		s1m2i1c2	1 (2.1)	1 (4.3)	
	s2	40 (51.9)	25 (59.5)			s1c2	15 (26.8)	3 (12)			s1m2i2c2	4 (8.5)	1 (4.3)	
***vacA* i**	i1	33 (44.6)	23 (62.2)	0.06		s2c1	0	5 (20)			s2m1i1c1	0	0	
	i2	41 (55.4)	14 (37.8)			s2c2	26 (46.4)	10 (40)			s2m1i1c2	0	1 (4.3)	
***vacA* m**	m1	31 (40.8)	21 (52.5)	0.22	***Vac* im**	i1m1	27 (38.6)	33 (61.1)	0.002		s2m1i2c1	0	1 (4.3)	
	m2	45 (59.2)	19 (47.5)			i2m1	2 (2.9)	2 (3.7)			s2m1i2c2	1 (2.1)	1 (4.3)	
***vacA* c**	c1	13 (27.1)	13 (50)	0.04		i1m2	5 (7.1)	9 (16.7)			s2m2i1c1	0	1 (4.3)	
	c2	35 (72.9)	13 (50)			i2m2	36 (51.4)	10 (18.5)			s2m2i1c2	1 (2.1)	1 (4.3)	
***cagA***	+	62 (80.5)	14 (31.8)	0.09	***vacA* ic**	i1c1	13 (27.7)	8 (34.8)	0.10		s2m2i2c1	0	1 (4.3)	
	−	15 (19.5)	30 (68.2)			i1c2	12 (25.5)	7 (30.4)			s2m2i2c2	22 (46.8)	7 (30.4)	
						i2c1	0	2 (8.7)						
***vacA* si**	s1i1	30 (40.5)	15 (40.5)			i2c2	22 (46.8)	6 (26.1)						
	s1i2	6 (8.1)	1 (2.7)	0.02	***vacA* mc**	m1c1	13 (27.1)	9 (36)	0.05					
	s2i1	3 (4.1)	8 (21.6)			m1c2	6 (12.5)	3 (12)						
	s2i2	35 (47.3)	13 (35.1)			m2c1	0	3 (12)						
						m2c2	29 (60.4)	10 (40)						

*Kruskall Wallis test.

**Table 4.** Simple logistic regression analysis of factors associated with GC

		Frequency (%)		Simple logistic regression		
		DU	GC	OR	95% CI	*P*-value
*vacA* c	c1	13 (27.1)	13 (50)	3.14	[1.08–9.09]	0.03
	c2	35 (72.9)	13 (50)	1 (ref)	1 (ref)	
Gender	Men	81 (73)	29 (59.2)	0.26	[0.08–0.79]	0.01
	Women	30 (27)	20 (40.8)	1 (ref)	1 (ref)	

DU, duodenal ulcer; GC, gastric cancer

## Discussion

Although *H. pylori* was first discovered in 1983 by Marshall and Warren [17], it remains a significant concern due to the serious health issues it can cause [18]. The severity of these disorders is influenced by various factors, particularly the bacterium’s virulence factors. Among the most studied is the *vacA* gene, which is present in all *H. pylori* strains and certain *vacA* genotypes are notably associated with an increased risk of developing GC. Several studies, including those conducted in Morocco, have investigated the frequency and correlation of different *vacA* genotypes (s, m, i), both individually and in combination, with gastric pathologies [6,8, 15, 19, 20]. In this study, we determined for the first time the distribution of the *vacA* c genotypes (c1 and c2) and the correlation of *vacA* s, m, i, c allelic combinations with gastroduodenal diseases among a Moroccan population.

The *vacA* gene of *H. pylori* contains several polymorphic regions, with the s, m, i and d regions being the most extensively studied. The allelic combinations of these regions vary across different geographical areas and may influence the toxicity of the VacA protein. In this study, molecular analysis revealed a predominance of the *vacA* c2 genotype [44.7% (317/709)] compared to the *vacA* c1 genotype [16.5% (117/709)]. These findings are consistent with previous studies that analysed other *vacA* regions, such as *vacA* m2 (57.6%), *vacA* s2 (50.2%) and *vacA* i2 (52%) [15], suggesting that less virulent genotypes are more common in our population. The predominance of the *vacA* c2 genotype has also been observed in Iran, where its prevalence ranges from 55.9 to 63.8% [11,21]. However, in the Northwestern province of Ardabil, *vacA* c1 was the most prevalent genotype, accounting for 53.7% of cases [22]. The heterogeneity in the distribution of *H. pylori* virulence genes may reflect regional differences in GC incidence. For instance, Ardabil, where the *vacA* c1 genotype is most prevalent, has been reported as the region with the highest GC incidence in Iran, with age-standardized rates of 51.8/100 000 in men and 24.9/100 000 in women [22]. Similarly, studies from Bangladesh, Egypt and Thailand have reported a predominance of the *vacA* c1 genotype, with prevalence rates of 53.6, 35.9 and 55.4%, respectively [23,25]. These findings further highlight the geographic variation in *H. pylori* genotype distribution and its potential link to GC risk.

The rate of MI [co-presence of both allelic forms of *vacA* c (*vacA* c1 and *vacA* c2 in the same samples)] in our study (9%) is similar to those reported in Iran (6.5%) but much lower than the 43.2% obtained in Thailand [21,25]. Also, the rate of non-genotyped *vacA* c region in our study (29.8%) was considerably lower than the 61.9% reported in Egypt but much higher than the 0% reported in Iran [21,24]. These differences in the non-detection rates could be attributed to the genetic variability of the *vacA* c region, which may vary by geographical region. The relatively high frequency of non-genotyped samples in our study may therefore be linked to the high polymorphism of the *vacA* c region. However, we also consider the possibility that the *vacA* c region may be absent in some strains, a hypothesis that will be further explored, especially since the primers used for genotyping the *vacA* c region were degenerated.

Few studies have investigated the recently identified polymorphic *vacA* c region of *H. pylori* and its association on one hand with other *vacA* allelic regions and on the other hand with gastric damage. This study reveals an equal distribution of the *vacA* c1 and *vacA* c2 *H. pylori* strains among patients with GC. Given that DU and GC were considered two opposite end outcomes of * H. pylori* infection [26], we used DU as the reference pathology in our logistic regression analysis. This analysis showed that *vacA* c1 *H. pylori* genotype is associated with an increased risk of GC [OR=3.14, CI 95% (1.08–9.09)] compared to the DU group. This result is consistent with the findings of Iranian studies where infection with *vacA* c1 *H. pylori* genotype has been linked to an increased risk of GC [OR=15.13, CI 95% (5.86–39.01)] and [OR=5.48, CI 95% (1.80–16.63)] when compared to the non-atrophic gastritis group [11,22]. However, the *H. pylori vacA* c2 predominance in gastroduodenal ulcers (65.7% in gastric ulcer cases and 72.9% in DU cases) is consistent with the results obtained in Bakhti *et al*.’s study (80.8% of *vacA* c2 in peptic ulcer disease patients vs. 19.2% of *vacA* c1) [11].

Several studies have reported that men were at higher risk to develop DU [27,28] and sometimes independently of the *H. pylori* infection. This was attributed to co-factors like smoking and hormones [29,32]. Other studies reported that men were more susceptible to develop severe diseases than women [15,19]. The same observation is reported in the present study, in which GC, GU (Gastric ulcer) and DU were predominantly present in men (59.2, 66.7 and 73% cases, respectively) than in women. So, when correlated to gender, *vacA* c1 was significantly predominant in strains infecting men (59.2%: 61/103 vs. 40.8%: 42/103 in women), while *vacA* c2 was frequently reported in strains infecting women (56.4%: 162/287 vs. 43.6% in men: 125/287). This susceptibility of men to infection with *H. pylori* that carries more virulent genotypes has also been reported in other previous studies [15,19]. Since *H. pylori* harbouring the *vacA* c1 allele is strongly associated with an increased risk of GC and is more commonly detected in men, it might suggest that men are at higher risk for developing GC. However, multivariate analysis shows that men are less susceptible to developing GC compared to DU [OR=0.26, CI 95% (0.08–0.79)]. This last result seems to be intriguing, but it may be explained by the following facts:

1. *vacA* c2 strains are more predominant than *vacA* c1 ones.
2. Patients carrying *H. pylori vacA* c1 genotype will not systematically develop GC but are at higher risk of developing it than the others. As the *vacA* gene harbours several regions whose combination appears to influence the degree of virulence, it’s more accurate to consider that *vacA* c (despite its significant association with certain pathologies) is not the sole factor affecting the progression of infection. In other words, the virulence of expressed *vacA* protein depends on the combined *vacA* alleles, and the *vacA* c region is likely just one piece of the puzzle.

In fact, despite the difference in the geographical distribution of the *vacA* allelic genotypes, most studies have shown a close association among the four regions of *vacA* [6,8, 33]. Our results show that *vacA* c1 is significantly predominant in strains carrying *vacA* s1m1i1 (67.8%), while *vacA* c2 is significantly predominant in strains carrying *vacA* s2m2i2 (60.9%). Those combinations could determine the *vacA* cytotoxicity level. In fact, previous *in vitro* studies have shown that the *vacA* s1m1i1 combination is more cytotoxic than the other combinations (*vacA* s1m1i2, s1m2i2 and s2m2i2) [6]. As in-depth studies of the *vacA* c region remain limited and sparse, its biological role is not yet clear. However, since the c1 genotype is reported in this and in another one to be more virulent than c2, its predominance in strains carrying the *vacA* s1m1i1 genotype (a combination already known to be virulent) suggests that the toxin derived from this *vacA* s1m1i1c1 genotype will also have high cytotoxic activity. Due to the limited and sparse nature of in-depth studies on the *vacA c* region, its biological function remains unclear. Nevertheless, the *vacA* c1 genotype has been reported to be more virulent than *vacA* c2 (the present study and Bakhti’s studies) [11,22]. Its prevalence in strains with the *vacA* s1m1i1 genotype, which is already recognized for its virulence, indicates that the toxin produced by the *vacA* s1m1i1c1 genotype is likely to exhibit significant cytotoxic activity.

*H. pylori* strains infecting our population were marked by a predominance of *vacA* s2m2i2c2 (27.2%), which is regarded as the less virulent pattern. This finding corroborates the conclusions reported in our previous studies and implies that less virulent genotypes are the most predominant in our population [12,15]. Nonetheless, our results diverged from those reported in an Iranian study where *vacA* s1m2i2d2c2 (37.9%) was the most prevalent, followed by *vacA* s1m2i2d2c2/*cagA*+ (21.6%), *vacA* s1m1i1d1c1 (17.9%) and *vacA* s1m1i1d1c1/*cagA* (15.8%) [21]. It’s also different from those reported in Bangladesh, Thailand and Egypt, which are marked by a predominance of *vacA* s1m1i1d1c1 (53.6, 32.4 and 22.8%, respectively) [23,25]. These data confirm the regional variation in the distribution of *H. pylori* strains.

Regarding the *vacA* combined genotypes and their associations with gastric pathologies, the results show that *vacA* s1m1i1c1 and *vacA* s2m2i2c2 were present with similar rates (30.4%) in GC cases, while other combinations, including rare genotypes, are present at a low rate (4.3%) with the exclusive presence of *vacA* s2m1i1c2, *vacA* s2m1i2c1, *vacA* s2m2i1c1 and *vacA* s2m2i2c1 in this malignant pathology. Even if this result was not statistically significant, it doesn’t support the previous studies indicating that strains carrying more virulent genotypes, like *vacA* s1m1i1c1, were predominantly associated with GC compared to *vacA* s2m2i2c2 genotype [21,23]. Moreover, in the univariate analysis, correlation of variables with pathologies (DU and GC) showed that genotypes *vacA* s1m1i2c1 and *vacA* s2m1i1c1 were not detected either in GC or in DU cases.

Additionally, the virulence of *H. pylori* was influenced by various other factors, including the presence of the *cagA* gene, which has been associated with the development of the most severe gastric diseases [34]. This correlation is supported by a significant association between *vacA* genotypes and *cagA* status. Specifically, it was found that 90.3% of *H. pylori vacA* c1 strains carried the *cagA* gene, while this gene was present in 62.8% of *H. pylori vacA* c2 strains.

In conclusion, our study confirms the considerable diversity of *H. pylori* strains circulating in our region with a predominance of the less virulent strains (*vacA* s2i2m2c2). Despite the unclear role of the *vacA* c region, the results of the present study suggest that the *vacA* c1 allele is a risk factor for the development of GC.

## Abbreviations

CI: confidence intervals
dNTP: deoxyribonucleotide triphosphate
DUs: duodenal ulcers
GC: gastric cancer
GU: gastric ulcer
MALT: Mucosa-Associated Lymphoid Tissue
MI: multiple infections
OR: odds ratio
